# Quercetin Inhibits the Production of IL-1β-Induced Inflammatory Cytokines and Chemokines in ARPE-19 Cells via the MAPK and NF-κB Signaling Pathways

**DOI:** 10.3390/ijms20122957

**Published:** 2019-06-17

**Authors:** Shu-Chen Cheng, Wen-Chung Huang, Jong-Hwei S. Pang, Yi-Hong Wu, Ching-Yi Cheng

**Affiliations:** 1 Department of Traditional Chinese Medicine, Chang Gung Memorial Hospital, Taoyuan 33372, Taiwan; kkaren0330@yahoo.com.tw; 2 Graduate Institute of Clinical Medical Sciences, College of Medicine, Chang Gung University, Taoyuan 33302, Taiwan; jonghwei@mail.cgu.edu.tw; 3 Graduate Institute of Health Industry Technology, Research Center for Chinese Herbal Medicine and Research Center for Food and Cosmetic Safety, College of Human Ecology, Chang Gung University of Science and Technology, Taoyuan 33303, Taiwan; wchuang@mail.cgust.edu.tw; 4 Division of Allergy, Asthma, and Rheumatology, Department of Pediatrics, Chang Gung Memorial Hospital, Taoyuan 33305, Taiwan; 5 Department of Physical Medicine and Rehabilitation, Chang Gung Memorial Hospital, Taoyuan 33305, Taiwan; 6 Division of Chinese Internal Medicine, Center for Traditional Chinese Medicine, Chang Gung Memorial Hospital, Taoyuan 33372, Taiwan; 7 School of Traditional Chinese Medicine, College of Medicine, Chang Gung University, Taoyuan 33302, Taiwan; 8 Department of Thoracic Medicine, Chang Gung Memorial Hospital, Linkou 33305, Taiwan; 9 Department of Ophthalmology, Chang Gung Memorial Hospital, Linkou 33305, Taiwan

**Keywords:** quercetin, retinal pigment epithelial cells, anti-inflammatory, cytokines, chemokines

## Abstract

Quercetin, a bioflavonoid derived from vegetables and fruits, exerts anti-inflammatory effects in various diseases. Our previous study revealed that quercetin could suppress the expression of matrix metalloprotease-9 (MMP-9) and intercellular adhesion molecule-1 (ICAM-1) to achieve anti-inflammatory effects in tumor necrosis factor-α (TNF-α)-stimulated human retinal pigment epithelial (ARPE-19) cells. The present study explored whether quercetin can inhibit the interleukin-1β (IL-1β)-induced production of inflammatory cytokines and chemokines in ARPE-19 cells. Prior to stimulation by IL-1β, ARPE-19 cells were pretreated with quercetin at various concentrations (2.5–20 µM). The results showed that quercetin could dose-dependently decrease the mRNA and protein levels of ICAM-1, IL-6, IL-8 and monocyte chemoattractant protein-1 (MCP-1). It also attenuated the adherence of the human monocytic leukemia cell line THP-1 to IL-1β-stimulated ARPE-19 cells. We also demonstrated that quercetin inhibited signaling pathways related to the inflammatory process, including phosphorylation of mitogen-activated protein kinases (MAPKs), inhibitor of nuclear factor κ-B kinase (IKK)α/β, c-Jun, cAMP response element-binding protein (CREB), activating transcription factor 2 (ATF2) and nuclear factor (NF)-κB p65, and blocked the translocation of NF-κB p65 into the nucleus. Furthermore, MAPK inhibitors including an extracellular signal-regulated kinase (ERK) 1/2 inhibitor (U0126), a p38 inhibitor (SB202190) and a c-Jun N-terminal kinase (JNK) inhibitor (SP600125) decreased the expression of soluble ICAM-1 (sICAM-1), but not ICAM-1. U0126 and SB202190 could inhibit the expression of IL-6, IL-8 and MCP-1, but SP600125 could not. An NF-κB inhibitor (Bay 11-7082) also reduced the expression of ICAM-1, sICAM-1, IL-6, IL-8 and MCP-1. Taken together, these results provide evidence that quercetin protects ARPE-19 cells from the IL-1β-stimulated increase in ICAM-1, sICAM-1, IL-6, IL-8 and MCP-1 production by blocking the activation of MAPK and NF-κB signaling pathways to ameliorate the inflammatory response.

## 1. Introduction

The retinal pigment epithelium (RPE), a single layer of cells located in the posterior part of the eye between the photoreceptors and vascularized choroid, is an indispensable part of the visual system and is responsible for several essential physiological functions. RPE cells can selectively transport nutrients and metabolic waste between the photoreceptors and choroid, maintain the ionic and fluid balance, absorb stray light, form the blood–retina barrier, phagocytose the photoreceptor outer segments, and secrete extracellular matrix components, hormones and growth factors for the photoreceptors, Bruch’s membrane and choriocapillaris [1,2,3]. When RPE cells are stimulated with inflammatory mediators such as tumor necrosis factor (TNF)-α, interferon-γ and interleukin-1β (IL-1β), they will produce cytokines and chemokines and then trigger inflammatory responses. Therefore, RPE cells are crucial elements in the pathogenesis of inflammation-associated progressive eye diseases, of which age-related macular degeneration (AMD) is the most important [4]

It is estimated that 8.7% of the global population suffer from AMD, and this number will probably double in the next 20 years with the increase in life expectancy. Consequently, AMD has become a major public health issue and an increased social and economic burden [5]. AMD is currently considered to be an irreversible permanent disease in the older population that is characterized by distorted central vision, a dark or gray patch (scotoma) in the central vision, and then progressive loss of central vision, which causes difficulties in daily living activities such as reading fine print or recognizing faces and color [6]. AMD has been classified into two distinct subtypes: Dry AMD (geographic atrophy; nonexudative) and wet AMD (neovascular; exudative), and its pathological processes includes lipofuscin accumulation, drusen formation, RPE geographic atrophy, photoreceptor dysfunction and degeneration, plus choroidal neovascularization [6,7,8]. Because of the elevated levels of inflammatory cytokines and chemokines such as IL-6, IL-8, intercellular adhesion molecule-1 (ICAM-1) and monocyte chemoattractant protein-1 (MCP-1), either locally in the ocular fluids or tissue or systemically in the serum of AMD patients, chronic inflammation is thought to facilitate the progress of AMD [9,10,11,12,13].

Quercetin is a natural bioflavonoid widely distributed in various vegetables and fruits such as onions, cranberries and green tea [14,15]. Quercetin possesses anti-inflammatory, anticarcinogenic, antioxidative, free-radical scavenging, antifibrotic and antiproliferative capacities that can be expressed in different cell types and animal models [15,16,17,18,19,20]. Based on these properties, quercetin has been documented to have the potential to treat diabetes [21], cancer [22,23], and neurodegenerative [24], liver [25] and cardiovascular diseases [26,27]. In ophthalmology, quercetin has recently been used to treat dry eye, corneal inflammation and corneal neovascularization [28]. Quercetin has also been reported to protect human retinal pigment epithelial (ARPE-19) cells against H_2_O_2_-induced injury [29], inhibit the expression of vascular cell adhesion molecule-1, ICAM-1, matrix metalloproteinase-2 (MMP-2) and MMP-9 in vascular endothelial growth factor-stimulated 661W cells [30], decrease TNF-α and IL-1β expression in rats with streptozotocin-induced diabetes [31], and reduce the production of IL-6, IL-8 and MCP-1 in 4-hydroxynonenal-stimulated ARPE-19 cells [15]. These studies provide a theoretical basis for the clinical application of quercetin in the prevention and treatment of retinal inflammatory diseases. However, the mechanisms by which quercetin mediates its anti-inflammatory effects are still debated.

In this study, we first investigated whether quercetin has anti-inflammatory properties in ARPE-19 cells stimulated by IL-1β and then analyzed the potential underlying pathways of inflammation. Understanding the role and mechanisms of action of quercetin could contribute to the discovery of effective therapeutic targets for retinal inflammatory diseases.

## 2. Results

### 2.1. IL-1β Induces the Expression of ICAM-1, sICAM-1, IL-6, IL-8 and MCP-1 in ARPE-19 Cells

**Figure 1:**
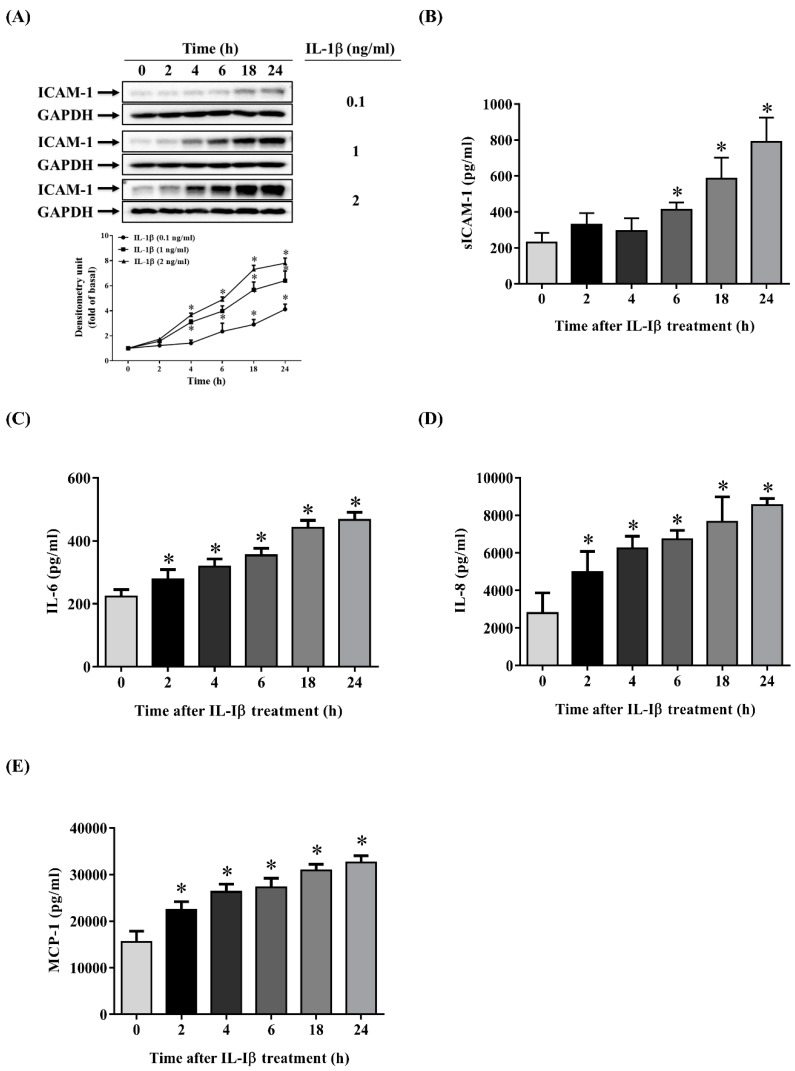
Interleukin-1β (IL-1β) induces the expression of intercellular adhesion molecule-1 (ICAM-1), soluble ICAM-1 (sICAM-1), IL-6, IL-8 and monocyte chemoattractant protein-1 (MCP-1) in human retinal pigment epithelial (ARPE-19) cells. (**A**) IL-1β at concentrations of 0.1–2 ng/mL was used to stimulate ARPE-19 cells for the indicated times. The protein expression of ICAM-1 was analyzed by Western blotting (top panels) and quantified by Image Lab software (lower panels). (**B**) The levels of sICAM-1, (**C**) IL-6, (**D**) IL-8 and (**E**) MCP-1 in ARPE-19 cells were measured using Enzyme-Linked Immunosorbent Assay (ELISA) after stimulation with 1 ng/mL IL-1β for the indicated times. The data are expressed as mean ± SD of three independent experiments. * p < 0.05 compared with the basal level.

The infiltration of macrophages or lymphocytes into the posterior chamber of the eye and the secretion of proinflammatory mediators such as IL-1β are important processes in retinal inflammation. IL-1β is an inducible proinflammatory cytokine that plays an early role in the production of inflammatory chemokines and cytokines. It triggers the inflammatory response and attracts more inflammatory cells to migrate into the retina, resulting in the functional impairment and degeneration of the retina. Therefore, ARPE-19 cells were treated for the specified time with or without various concentrations (0.1, 1, 2 ng/mL) of IL-1β, to explore whether the production of ICAM-1, sICAM-1, IL-6, IL-8 and MCP increased after this stimulation. The concentrations of the IL-1β (0.1, 1, 2 ng/mL) used alone had no toxic effects or changes in the cell viability on ARPE-19 cells, as tested for an LDH release test or a 3-(4,5-dimethylthiazol-2-yl)-2,5-diphenyltetrazolium bromide (MTT) assay (data not shown). As shown in Figure 1A–E, the increases in the levels of ICAM-1, sICAM-1, IL-6, IL-8 and MCP-1 detected by Enzyme-Linked Immunosorbent Assay (ELISA) or Western blotting were positively correlated with the concentration of IL-1β and the stimulation time.

### 2.2. Quercetin Inhibits the Expression of ICAM-1, sICAM-1, IL-6, IL-8 and MCP-1 in IL-1β-Stimulated ARPE-19 Cells

**Figure 2:**
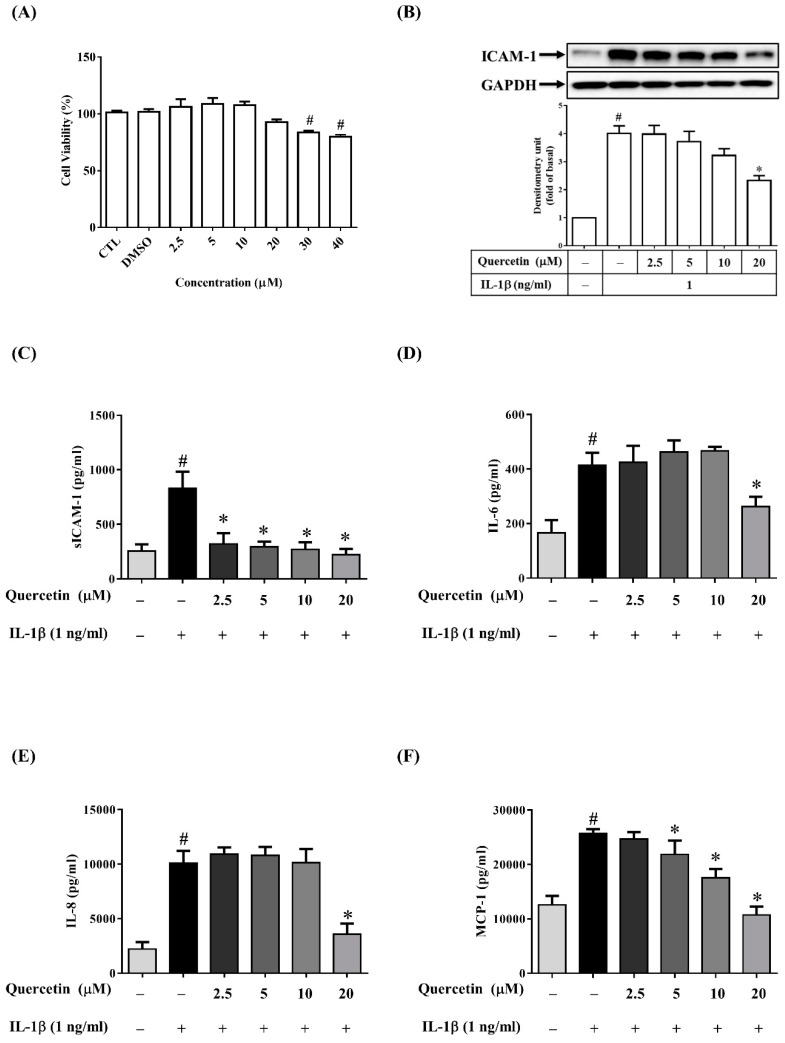
Quercetin attenuates the expression of ICAM-1, sICAM-1, IL-6, IL-8 and MCP-1 in IL-1β-stimulated ARPE-19 cells. (**A**) Effects of quercetin on ARPE-19 cell viability. ARPE-19 cells were treated for 24 h with 2.5–40 µM quercetin and a 3-(4,5-dimethylthiazol-2-yl)-2,5-diphenyltetrazolium bromide (MTT) assay was used to analyze the cell viability. (**B**) ICAM-1 protein level was evaluated by Western blotting and then quantified using Image Lab software. (**C**) The levels of sICAM-1, (**D**) IL-6, (**E**) IL-8 and (**F**) MCP-1 were assessed by ELISA after cells were incubated for 1 h with quercetin at the indicated doses and then activated with 1 ng/mL IL-1β for 24 h. The data are expressed as mean ± SD of three independent experiments. ^#^ *p* < 0.05 versus control cells. * *p* < 0.05 versus IL-1β-stimulated cells.

**Figure 3:**
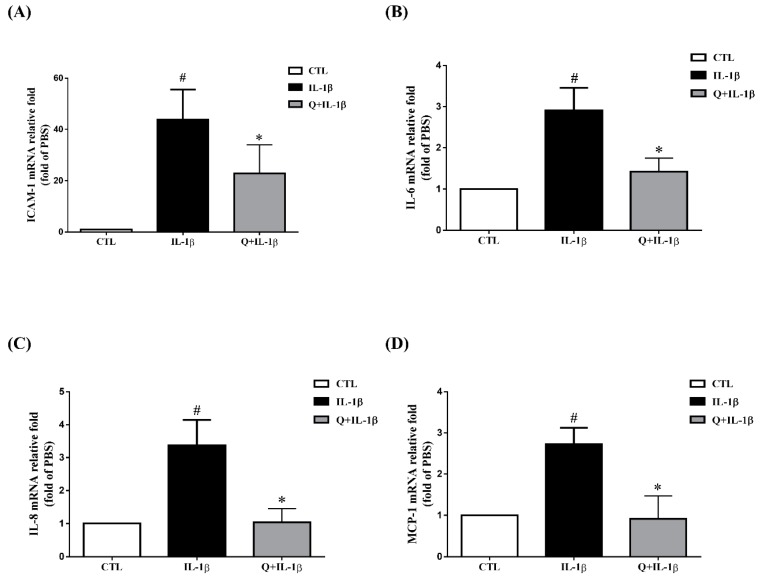
Quercetin attenuates the expression of ICAM-1, IL-6, IL-8 and MCP-1 mRNA in IL-1β-stimulated ARPE-19 cells. ARPE-19 cells were pretreated with 20 µM quercetin for 1 h before stimulation with 1 ng/mL IL-1β for 4 h. Reverse Transcription-Quantitative Polymerase Chain Reaction (RT-qPCR) was used to determine the fold changes in (**A**) ICAM-1, (**B**) IL-6, (**C**) IL-8 and (**D**) MCP-1 gene expression with β-actin as an internal control. The data are expressed as mean ± SD of three independent experiments. ^#^ *p* < 0.05 versus control cells. * *p* < 0.05 versus IL-1β-stimulated cells.

Numerous studies have reported the quercetin can inhibit the expression of IL-6, IL-8, ICAM-1 or MCP-1 induced by various stimuli such as LPS, TNF-α, high glucose and calcium ionophore A23187 in human mast cells, mesangial cells, neutrophils, airway epithelial cells and rat intestinal microvascular endothelial cells, respectively [32,33,34,35,36]. In these experiments, the efficacy and modes of action of quercetin appear to be affected by a diversity of cell types and inflammatory stimulants. Therefore, we evaluated whether quercetin has anti-inflammatory properties in IL-1β-stimulated ARPE-19 cells. We first assessed the cytotoxicity of quercetin in ARPE-19 cells by an MTT assay. As shown in Figure 2A, the viability of ARPE-19 cells was significantly reduced at quercetin concentrations higher than 30 µM. Accordingly, quercetin concentrations from 2.5 to 20 μM were chosen for all subsequent experiments (ELISA, Western blotting, and Reverse Transcription-Quantitative Polymerase Chain Reaction (RT-qPCR) tests). Before being stimulated with 1 ng/mL IL-1β for 24 h, ARPE-19 cells were pretreated with different concentrations of quercetin (2.5, 5, 10 or 20 µM) for 1 h. As the quercetin concentration increased, the ICAM-1 level gradually decreased and the release of sICAM-1 into the culture medium was inhibited (Figure 2B,C). Twenty micromolar quercetin also significantly inhibited the expression of IL-6, IL-8 and MCP-1 (Figure 2D–F). To investigate whether quercetin affects the mRNA expression of ICAM-1, IL-6, IL-8 and MCP-1 in IL-1β-stimulated ARPE-19 cells, cells were pretreated with 20 µM quercetin for 1 h and then incubated with IL-1β (1 ng/mL) for 4 h. Quercetin clearly reduced the IL-1β-induced expression of mRNA for ICAM-1, IL-6, IL-8 and MCP-1 (Figure 3A–D).

### 2.3. Quercetin Suppresses Inflammatory Signaling Pathways in ARPE-19 Cells

**Figure 4:**
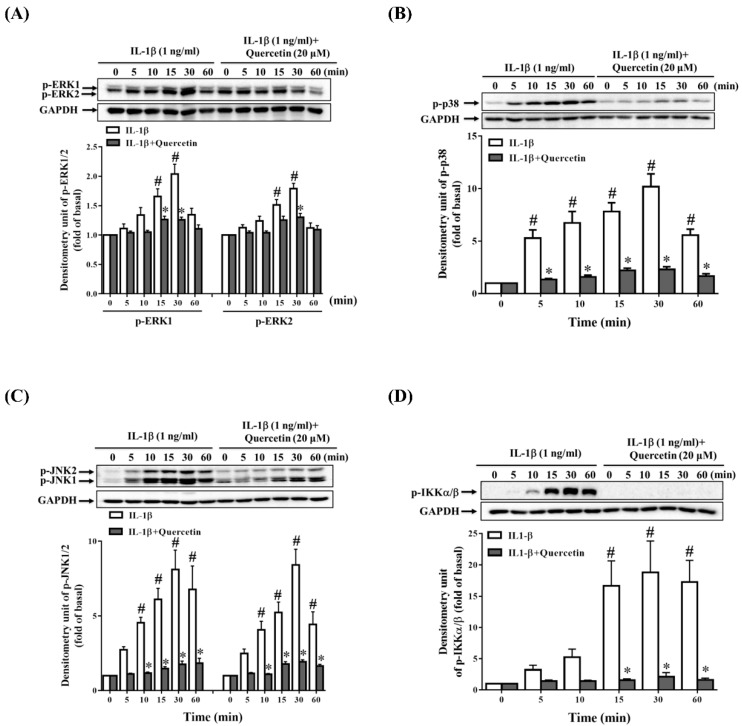
Quercetin inhibits the phosphorylation of mitogen-activated protein kinases (MAPKs) and inhibitor of nuclear factor κ-B kinase (IKK)α/β in IL-1β-stimulated ARPE-19 cells. ARPE-19 cells were treated with 20 µM quercetin for 1 h prior to the stimulation with 1 ng/mL IL-1β for the indicated time. Western blotting and Image Lab software were used to analyze and quantify the phosphorylation of (**A**) extracellular signal-regulated kinase (ERK) 1/2, (**B**) p38, (**C**) c-Jun N-terminal kinase (JNK) 1/2 and (**D**) IKKα/β. The data are expressed as mean ± SD of three independent experiments. ^#^ *p* < 0.05 versus control cells. * *p* < 0.05 versus IL-1β-stimulated cells.

**Figure 5:**
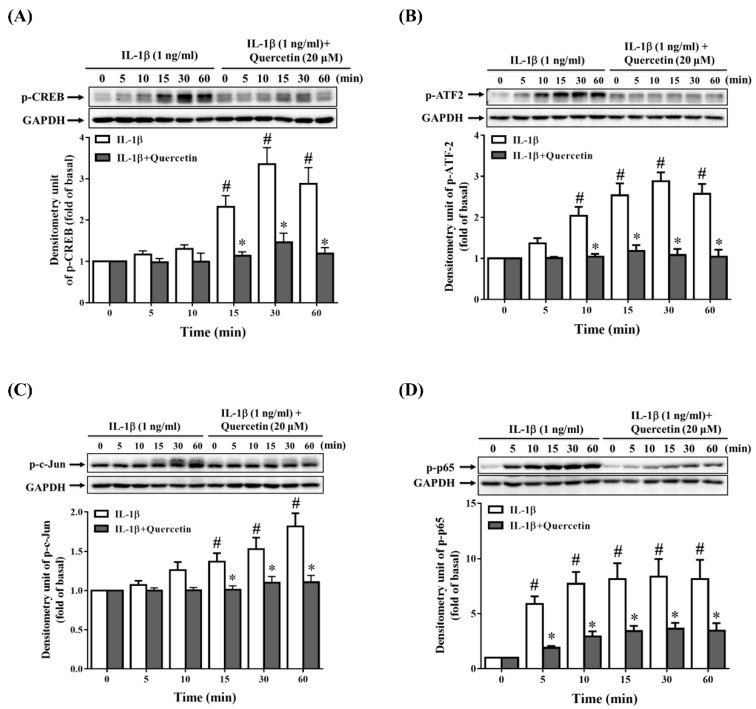
Quercetin attenuates the phosphorylation of cAMP response element-binding protein (CREB), activating transcription factor 2 (ATF2), c-Jun and nuclear factor (NF)-κB p65 in IL-1β-stimulated ARPE-19 cells. ARPE-19 cells were treated with 20 µM quercetin for 1 h prior to stimulation with 1 ng/mL IL-1β for the indicated time. Western blotting and Image Lab software were used to analyze and quantify the phosphorylation of (**A**) CREB, (**B**) ATF2, (**C**) c-Jun and (**D**) Nuclear factor (NF)-κB p65. The data are expressed as mean ± SD of three independent experiments. ^#^ *p* < 0.05 versus control cells. * *p* < 0.05 versus IL-1β-stimulated cells.

Many studies have demonstrated that quercetin can combat inflammation through regulating mitogen-activated protein kinase (MAPK) pathways in different types of cells under different stimulants [14,32,36,37,38]. Because we observed that quercetin inhibited the expression of ICAM-1, sICAM-1, IL-6, IL-8 and MCP-1 in IL-1β-stimulated ARPE-19 cells, we investigated whether quercetin could suppress the phosphorylation of signaling pathway proteins in these cells. The cells were incubated with 20 µM quercetin for 1 h before stimulation with 1 ng/mL IL-1β for the indicated times. The results demonstrated that quercetin significantly attenuated the phosphorylation of MAPKs (extracellular signal-regulated kinase (ERK) 1/2, p38, and c-Jun N-terminal kinase (JNK) 1/2), cAMP response element-binding protein (CREB), activating transcription factor 2 (ATF2) and c-Jun in IL-1β-stimulated ARPE-19 cells, implying that these proteins may promote the production of ICAM-1, sICAM-1, IL-6, IL-8 and MCP-1 (Figure 4A–C and Figure 5A–C).

### 2.4. MAPK Inhibitors Decrease the IL-1β-Induced Expression of sICAM-1, IL-6, IL-8 and MCP-1 in ARPE-19 Cells

**Figure 6:**
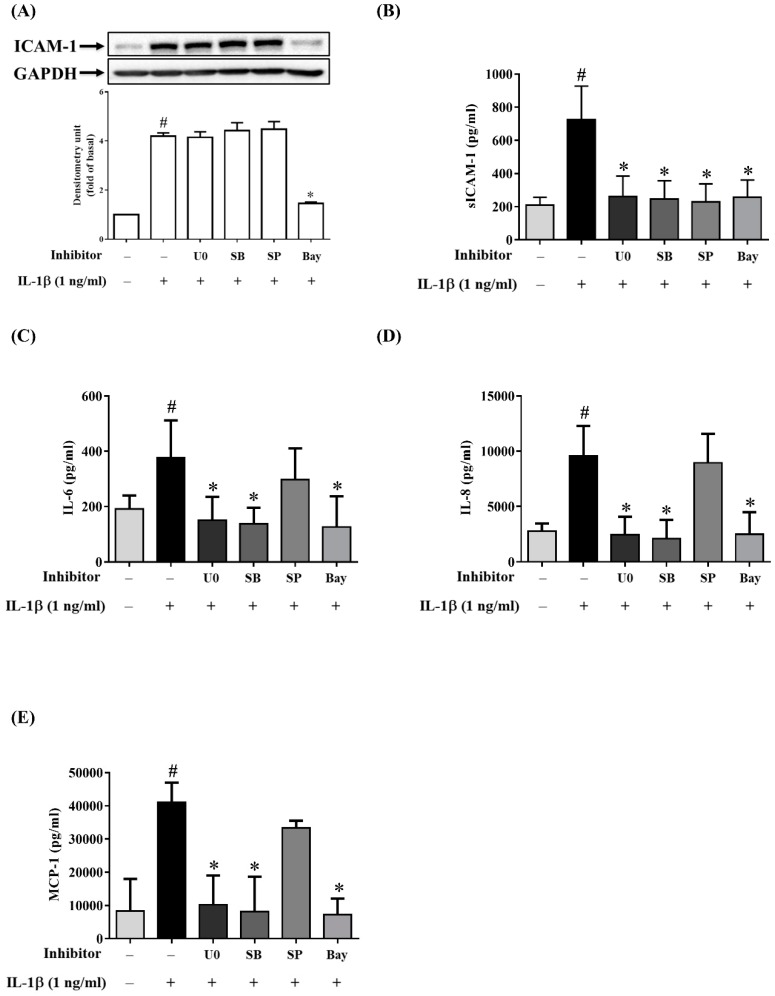
Inhibitory effects of MAPKs and NF-κB inhibitors on the protein expression of ICAM-1, sICAM-1, IL-6, IL-8 and MCP-1 in IL-1β-stimulated ARPE-19 cells. ARPE-19 cells were pretreated with 10 µM U0126 (U0), 10 µM SB202190 (SB), 10 µM SP600125 (SP) or 5 µM Bay11-7082 (Bay) for 1 h prior to stimulation with 1 ng/mL IL-1β for 24 h. (**A**) ICAM-1 protein expression was measured by Western blotting and quantified using Image Lab software. (**B**) The levels of sICAM-1, (**C**) IL-6, (**D**) IL-8 and (**E**) MCP-1 were detected by ELISA. The data are expressed as mean ± SD of three independent experiments. ^#^ *p* < 0.05 versus control cells. * *p* < 0.05 versus IL-1β-stimulated cells.

**Figure 7:**
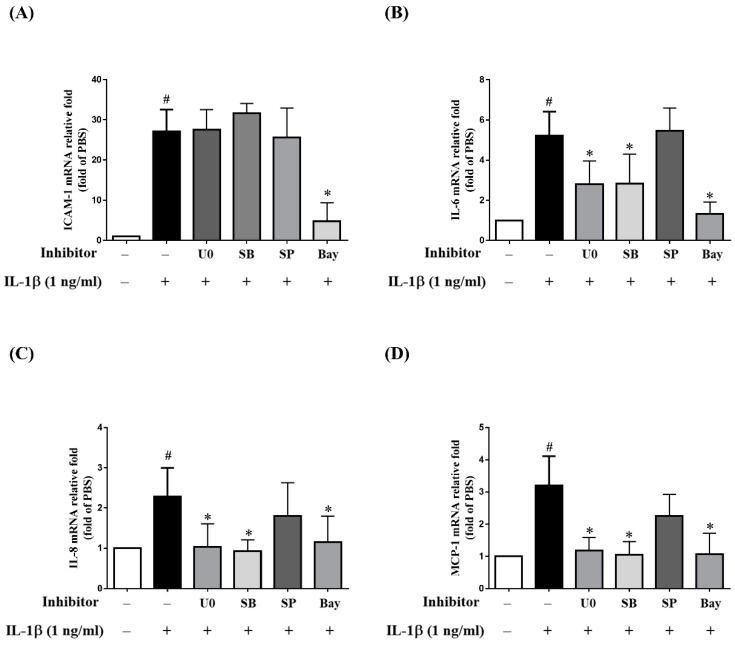
Inhibitory effects of MAPKs and NF-κB inhibitors on the expression of mRNA for ICAM-1, IL-6, IL-8 and MCP-1 in IL-1β-stimulated ARPE-19 cells. ARPE-19 cells were treated with 10 µM U0126 (U0), 10 µM SB202190 (SB), 10 µM SP600125 (SP) or 5 µM Bay11-7082 (Bay) for 1 h, followed by stimulation with 1 ng/mL IL-1β for 4 h. The fold changes in (**A**) ICAM-1, (**B**) IL-6, (**C**) IL-8 and (**D**) MCP-1 gene expression were analyzed using RT-qPCR with β-actin as an internal control. The data are expressed as mean ± SD of three independent experiments. ^#^ *p* < 0.05 versus control cells. * *p* < 0.05 versus IL-1β-stimulated cells.

While previous studies have reported the potential roles of MAPKs in RPE cells treated with different stimulants and inhibitors, the results are inconsistent [39,40,41,42]. To explore the importance of three separate MAPKs in retinal inflammatory diseases, ARPE-19 cells were pretreated with MAPK inhibitors (10 µM p38 inhibitor SB202190, 10 µM ERK1/2 inhibitor U0126 or 10 µM JNK inhibitor SP600125) for 1 h prior to incubation with 1 ng/mL IL-1β for 24 h. Interestingly, the MAPK inhibitors reduced the IL-1β-induced expression of sICAM-1 but not that for ICAM-1 and ICAM-1 mRNA levels (Figure 6A,B and Figure 7A). Next, we investigated whether MAPK inhibitors could attenuate the IL-1β-stimulated production of inflammatory cytokines IL-6, IL-8 and MCP-1. As shown in Figure 6C–E, the release of IL-6, IL-8 and MCP-1 was reduced by U0126 and SB202190, but not by SP600125. Similar trends were observed for the expression of mRNA for IL-6, IL-8 and MCP-1 (Figure 7B–D). These results suggested that in IL-1β-stimulated ARPE-19 cells, quercetin reduces sICAM-1 levels via the p38, ERK1/2 and JNK1/2 pathways and suppresses IL-6, IL-8 and MCP-1 levels via the p38 and ERK1/2 pathways.

### 2.5. Quercetin Decreases Nuclear Factor (NF)-κB Activation in IL-1β-Stimulated ARPE-19 Cells

Previous studies have confirmed that NF-κB plays an indispensable role in inflammation [43]. When RPE cells are stimulated by proinflammatory cytokines such as IL-1β, phosphorylation of the inhibitor of NF-κB (IκB) is induced, leading to translocation of NF-κB into the nucleus, which results in the transcription of cytokine and chemokine genes. Quercetin has been demonstrated to exert its anti-inflammatory effects through downregulating the NF-κB signaling pathways in vitro [44,45,46] and in vivo [47,48]. In the present study, ARPE-19 cells were pretreated with 20 µM quercetin for 1 h prior to the stimulation with IL-1β (1 ng/mL) for the indicated times to investigate whether quercetin could reduce the phosphorylation of inhibitor of nuclear factor κ-B kinase (IKK)α/β and NF-κB p65. As shown in Figure 4D and Figure 5D, quercetin clearly suppressed the IL-1β-induced phosphorylation of IKKα/β and NF-κB p65 in ARPE-19 cells.

We next explored the role of NF-κB in the expression of ICAM-1, sICAM-1, IL-6, IL-8 and MCP-1 in IL-1β-stimulated ARPE-19 cells. As shown in Figure 6A,B, when cells were pretreated with 5 µM Bay11-7082, the expression of both sICAM-1 and ICAM-1 was decreased. Pretreatment with Bay11-7082 also downregulated ICAM-1 mRNA expression (Figure 7A). Similar outcomes were also obtained for the expression of protein and mRNA for IL-6, IL-8 and MCP-1 (Figure 6C–E and Figure 7B–D). These results suggested that NF-κB is the principal pathway mediating the reduction of ICAM-1, sICAM-1, IL-6, IL-8 and MCP-1 levels in IL-1β-stimulated ARPE-19 cells.

**Figure 8:**
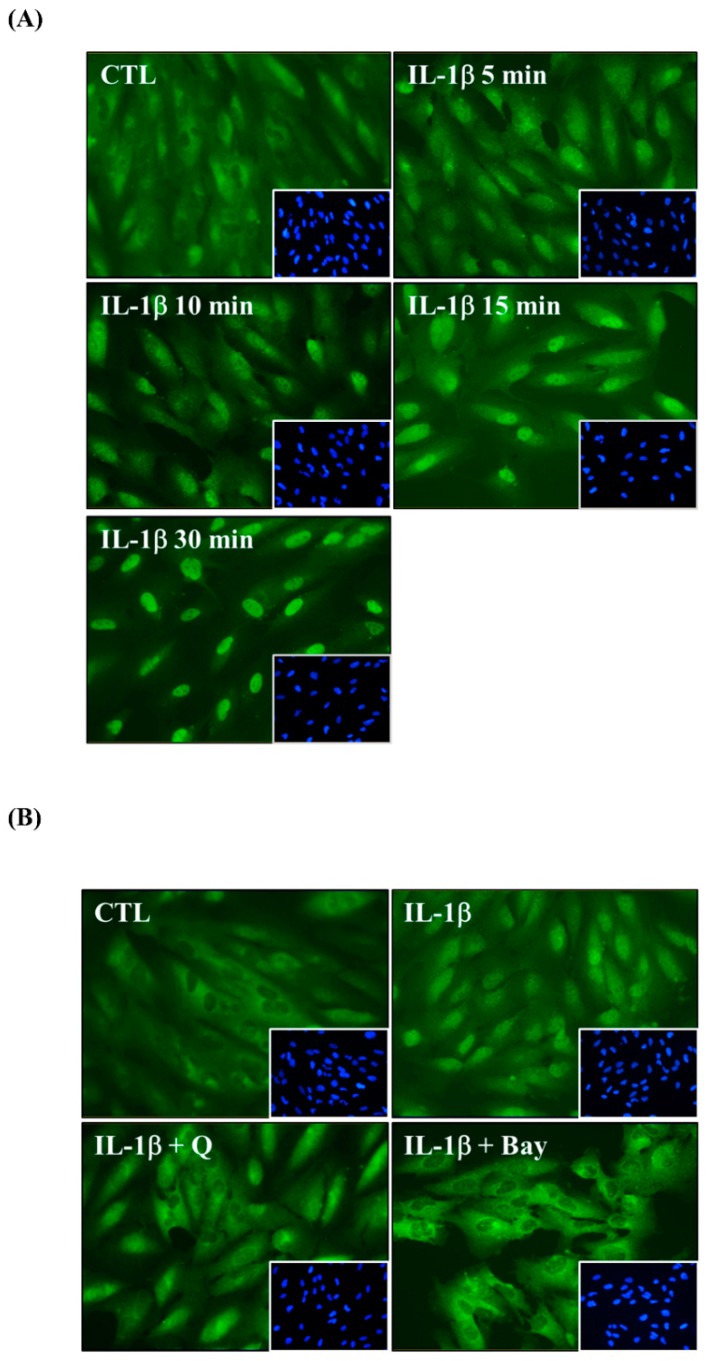
Quercetin attenuates NF-κB p65 translocation in IL-1β-stimulated ARPE-19 cells. Immunofluorescence staining was used to evaluate NF-κB p65 translocation in (**A**) ARPE-19 cells stimulated with 1 ng/mL IL-1β for the indicated time, and (**B**) ARPE-19 cells pretreated with 20 µM quercetin or 5 µM Bay 11-7082 for 1 h prior to activation with 1 ng/mL IL-1β for 10 min. The image is representative of the results of four independent experiments. *Green*: the location of the p65 subunit; *Blue*: DAPI for nuclear staining.

Immunofluorescence staining was also used to elucidate whether quercetin attenuated IL-1β-induced NF-κB p65 translocation from the cytoplasm into the nucleus. First, we used 1 ng/mL IL-1β to stimulate ARPE-19 cells for the indicated times. Although the NF-κB p65 subunit was mainly present in the cytoplasm in the unstimulated ARPE-19 cells, the results indicated that IL-1β induced NF-κB p65 translocation within 5 min and achieved the maximal response within 30 min (Figure 8A). Pretreatment of cells with either quercetin or Bay 11-7082 before their stimulation with IL-1β blocked NF-κB p65 translocation into the nucleus so that the p65 subunit was retained in the cytoplasm (Figure 8B). These results suggested that quercetin attenuated the expression of ICAM-1, sICAM-1, IL-6, IL-8 and MCP-1 in IL-1β-stimulated ARPE-19 cells by downregulating NF-κB p65 translocation.

### 2.6. Quercetin Attenuates THP-1 Cell Adherence to IL-1β-Stimulated ARPE-19 Cells

**Figure 9:**
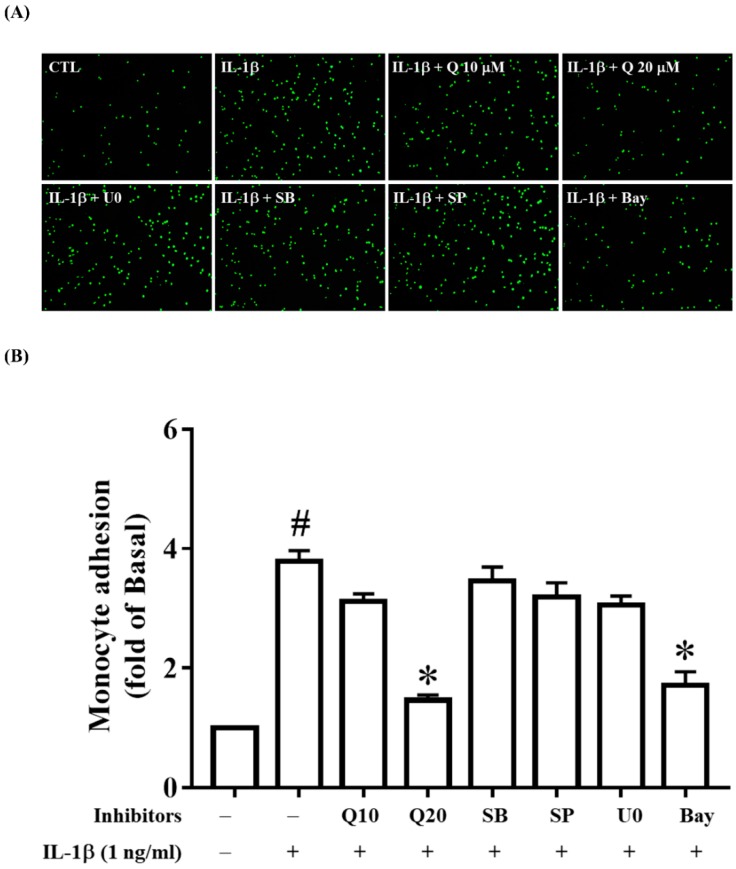
Quercetin significantly suppresses THP-1 cell adherence to IL-1β-stimulated ARPE-19 cells. ARPE-19 cells were preincubated with quercetin (10, 20 µM), 10 µM U0126, 10 µM SB202190, 10 µM SP600125 or 5 µM Bay 11-7082 for 1 h and then stimulated with 1 ng/mL IL-1β for 24 h. (**A**,**B**) A THP-1 monocyte adhesion assay was used to evaluate the physiological function of ICAM-1. The fluorescence intensity represents THP-1 cell adhesion to IL-1β-stimulated ARPE-19 cells, which was quantified using Image J software. The data are expressed as mean ± SD of three independent experiments. ^#^ *p* < 0.05 versus control cells. * *p* < 0.05 versus IL-1β-stimulated cells.

It has been reported that decreased ICAM-1 expression results in the suppression of the adhesion of THP-1 cells [41,49]. Because we demonstrated that quercetin strongly inhibited ICAM-1 expression, we wanted to investigate whether quercetin could also attenuate THP-1 cell adhesion to IL-1β-stimulated ARPE-19 cells. Indeed, pretreatment with 20 µM quercetin seemed to significantly decrease THP-1 cell adherence to IL-1β-activated ARPE-19 cells (Figure 9A,B). We next investigated whether the inhibitors of ERK1/2, p38, JNK1/2 and NF-κB could modulate THP-1 cell adhesion and showed that NF-κB inhibitor (5 µM Bay 11-7082) significantly attenuated THP-1 cell adhesion to IL-1β-stimulated ARPE-19 cells (Figure 9A,B). Combined with the fact that quercetin inhibited the phosphorylation of IKKα/β and NF-κB p65 and blocked NF-κB p65 translocation, these findings illustrate that quercetin decreased ICAM-1 expression via the NF-κB pathway, which contributed to the enhancement of THP-1 cell adhesion to IL-1β-stimulated ARPE-19 cells.

## 3. Discussion

Inflammation has been reported to be involved in the pathophysiology of various retinal diseases, including AMD, polypoidal choroidal vasculopathy, diabetic retinopathy and retinal vein occlusion [50,51,52,53,54]. RPE cells have been demonstrated to secrete cytokines in vitro after stimulation with IL-1β [55]. IL-1β is a proinflammatory cytokine that can trigger the inflammatory cascade and plays a major role in retinal inflammation [54,56]. Previous studies have also shown that IL-1β upregulates the expression of IL-6 [55,57,58], IL-8 [55,59], ICAM-1 [60] and MCP-1 [39,61] in human RPE cells. In the present study, stimulation of ARPE-19 cells with IL-1β induced increased production of ICAM-1, IL-6, IL-8 and MCP-1, which is consistent with these previous results.

ICAM-1, also known as CD54, is a transmembrane glycoprotein that plays a key role in recruitment, adhesion and infiltration of neutrophils and monocytes to the retina [62,63,64]. The excessive proinflammatory cytokines released by these neutrophils or monocytes initiate inflammation and aggravate damage. Previous studies have indicated that ICAM-1 expression is increased in posterior uveitis, proliferative vitreoretinopathy, proliferative diabetic retinopathy and AMD [12,60,64]. The sICAM-1 detected in culture supernatants and human body fluids such as serum, synovial fluid and urine has been documented to recruit lymphocytes and eosinophils to inflamed tissue [65,66]. Nevertheless, the mechanisms involved in sICAM-1 generation have not been fully elucidated. It is thought that sICAM-1 is either produced by proteolytic cleavage of membrane-bound ICAM-1 or is specifically encoded by distinct mRNA transcripts [67,68]. Previous studies have confirmed that sICAM-1 levels are elevated in patients with proliferative retinal disease [69], Graves’ ophthalmopathy [70], idiopathic uveoretinitis [71] and various inflammatory diseases, and that sICAM-1 levels could be used to assess illness severity and prognosis [72,73,74,75,76]. IL-6, a multifunctional cytokine, contributes to activating T lymphocytes, stimulating immunoglobulin secretion, increasing vascular permeability and triggering acute-phase protein release [77,78,79]. MCP-1 (also called CCL2) belongs to the C–C chemokine family and stimulates and attracts monocytes and lymphocytes, resulting in monocyte/macrophage infiltration [80,81]. IL-8 belongs to the C–X–C chemokine family and is a chemoattractant for eosinophils and neutrophils [11]. Previous studies have demonstrated that IL-6, IL-8 and MCP-1 not only initiate inflammatory responses but also promote angiogenesis, thereby stimulating AMD progression [10,82,83,84,85]. Our results showed that the levels of ICAM-1, sICAM-1, IL-6, IL-8 and MCP-1 in IL-1β-stimulated ARPE-19 cells were positively correlated with the IL-1β concentration and the duration of stimulation, suggesting that these cytokines and chemokines play a crucial part in the process of RPE inflammation.

Previous ophthalmic studies suggested that quercetin reduced IL-6 and IL-8 mRNA expression in cultured tissue from Graves’ orbitopathy [16], attenuated IL-6, IL-8 and ICAM-1 mRNA levels in IL-1β-stimulated orbital fibroblasts from Graves’ orbitopathy [86], and inhibited IL-6 and IL-8 secretion in TNF-α-stimulated human corneal epithelial (HCE) and conjunctival (IOBA-NHC) cell lines [87]. In addition, quercetin was shown to attenuate TNF-α-induced ICAM-1 and MMP-9 expression in ARPE-19 cells [88], and reduce both the RNA and protein levels of IL-6, IL-8 and MCP-1 in 4-hydroxynonenal-stimulated ARPE-19 cells [15]. Thus, there is increasing evidence that quercetin may protect RPE cells from damage in vitro [15,88,89,90]. The present study provides the first evidence that quercetin inhibits the mRNA and protein expression of IL-6, IL-8, MCP-1, ICAM-1 and sICAM-1 in IL-1β-stimulated ARPE-19 cells. We also showed that quercetin suppressed THP-1 cell adherence to IL-1β-stimulated ARPE-19 cells. These findings suggest that quercetin could have anti-inflammatory activity in IL-1β-stimulated ARPE-19 cells.

Because inflammation may be a key factor in RPE degeneration, dysfunction and loss in retinal degenerative diseases, the intracellular signaling pathways involved in initiating the release of cytokines and chemokines in RPE cells are important. One of the most widely reported signaling pathways in many cell systems is the MAPK signaling pathway, in which inflammatory stimulants contribute to the activation of MAPKs, followed by increased release of cytokines and chemokines [91,92,93]. Quercetin has been reported to have anti-inflammatory effects via inhibiting the activation of MAPKs in a number of different cell lines treated with different inflammatory stimulants [14,34,36,37]. In this study, we illustrated that IL-1β activated the phosphorylation of MAPKs (ERK1/2, p38 and JNK1/2), c-Jun and transcription factors (CREB and ATF2) in ARPE-19 cells, and that quercetin significantly suppressed this phosphorylation, which in turn led to a reduction in the expression of ICAM-1, sICAM-1, IL-6, IL-8 and MCP-1.

We also used MAPK-inhibitor treatment of IL-1β-stimulated ARPE-19 cells to explore the significance of individual MAPKs. We discovered that MAPK inhibitors, including SB202190, SP600125 and U0126, did not reduce the expression of ICAM-1, but did reduce that of sICAM-1. These findings indicated that ICAM-1 and sICAM-1 were regulated by different signaling mechanisms in IL-1β-stimulated ARPE-19 cells. We also showed that although ERK1/2 and p38 inhibitors suppressed the expression of IL-6, IL-8 and MCP-1, JNK inhibitor did not. Some of these observations are identical to the findings reported by Bian et al. who showed that only ERK1/2 or p38 inhibitors were able to reduce IL-8 and MCP-1 levels in IL-1β-stimulated ARPE-19 cells [39].

There is increasing evidence that NF-κB influences the inflammatory process by regulating the gene and protein expression of cytokines and chemokines [78]. The inflammatory signals generated by the stimulation of ARPE-19 cells by IL-1β induce the production of phosphorylated IKKα/β and lead to the activation of NF-κB. The activated NF-κB then moves into the nucleus from the cytoplasm, resulting in inflammatory gene expression. Many studies have reported that quercetin exerts its anti-inflammatory effects mainly through downregulation of NF-κB [15,30,32,94].

Our previous studies found that quercetin decreased ICAM-1 expression by downregulating NF-κB in TNF-α-stimulated ARPE-19 cells [88]. In the present study, we demonstrated that quercetin significantly inhibited phosphorylation of IKKα/β and NF-κB p65 and reduced NF-κB p65 translocation into the nucleus. IL-1β-stimulated ARPE-19 cells were treated with NF-κB inhibitor (Bay 11-7082) to determine whether quercetin downregulated NF-κB activation and thereby attenuated the mRNA and protein levels of ICAM, sICAM-1, IL-6, IL-8 and MCP-1. We observed that Bay11-7082 decreased the expression of ICAM, sICAM-1, IL-6, IL-8 and MCP-1 and that this inhibition was associated with the downregulation of the NF-κB signaling pathway. Thus, we confirmed that NF-κB has an effect on the regulation of cytokine and chemokine production in these cells.

Previous studies have shown that ICAM-1 is involved in the recruitment of monocytes, neutrophils and lymphocytes, and in the adhesive interactions of THP-1 cells [95,96,97]. In this study, we investigated whether quercetin reduced ICAM-1 levels and hence affected THP-1 cell adhesion to ARPE-19 cells. We found that 20 µM quercetin reduced the expression of ICAM-1 in ARPE-19 cells and inhibited the adhesion of THP-1 cells to IL-1β-stimulated ARPE-19 cells. We also observed that the ICAM-1 level was regulated only by the NF-κB pathway, and not by the MAPK pathway: When inhibitors of MAPKs or NF-κB were used to treat IL-1β-stimulated ARPE-19 cells, only the NF-κB inhibitor reduced THP-1 cell adhesion. Taken together, these observations indicated that quercetin downregulates the NF-κB pathway to decrease the ICAM-1 level and thereby inhibits THP-1 cell adhesion to ARPE-19 cells.

## 4. Materials and Methods

### 4.1. Materials

Anti-phospho-ATF-2, anti-phospho-c-Jun, anti-phospho-CREB, anti-phospho-Erk1/2, anti-phospho-IKKα/β, anti-phospho-JNK1/2, anti-phospho-p38 and anti-phospho-NF-κB p65 antibodies were obtained from Cell Signaling Technology (Danvers, MA, USA). Anti-ICAM-1, anti-glyceraldehyde 3-phosphate dehydrogenase (GAPDH) and anti-NF-κB p65 antibodies were obtained from Santa Cruz Biotechnology (Santa Cruz, CA, USA). Bay 11-7082, SB202190, SP600125 and U0126 were obtained from Enzo Life Sciences (Farmingdale, NY, USA). Human recombinant IL-1β was obtained from R&D Systems (Minneapolis, MN, USA). Quercetin was obtained from HWI Analytik (Rheinzabern, Germany). Quercetin stock solution was dissolved in dimethyl sulfoxide (DMSO) and then diluted to the desired concentrations with culture medium. All other reagents used in the experiments were obtained from Sigma-Aldrich (St. Louis, MO, USA).

### 4.2. Cell Culture

The human retinal pigment epithelial cell line, ARPE-19 cells (Bioresource Collection and Research Center, Hsinchu City, Taiwan), was cultured in Dulbecco’s modified Eagle’s medium (DMEM)/F-12 medium (Gibco BRL, Grand Island, NY, USA) containing sodium bicarbonate, 10% (*v*/*v*) fetal bovine serum (FBS; HyClone, Logan, UT, USA) and antibiotics (50 ng/mL gentamycin, 100 U/mL penicillin G and 100 µg/mL streptomycin (HyClone). Cells were subcultured every 3 to 4 days using 0.05% (*v*/*v*) trypsin-ethylenediaminetetraacetic acid (EDTA; Life Technologies, Carlsbad, CA, USA).

The human monocytic leukemia cell line (THP-1 cells) was obtained from the American Type Culture Collection (Manassas, VA, USA) and grown in RPMI 1640 medium (Gibco) containing antibiotics and 10% FBS in a humidified 5% CO_2_ atmosphere at 37 °C. We changed the medium every 4 to 5 days.

### 4.3. Cell Viability Assay

The MTT (Sigma-Aldrich) assay was used to measure the inhibition of cell viability by quercetin. Cells were seeded into 96-well plates and treated with quercetin at different concentrations (2.5–40 µM) for 24 h. Next, each well was incubated with 0.5 mg/mL MTT solution for 1 h at 37 °C. The plates were then washed and DMSO added to dissolve the formazan crystals followed by analysis using a SpectraMax i3x microplate reader (Molecular Devices, San Jose, CA, USA) at 570 nm. The MTT assay for each concentration was carried out in triplicate and the cell viability is presented as a percentage relative to the cells without quercetin treatment.

### 4.4. ELISA

ARPE-19 cells were pretreated with or without quercetin (2.5–20 µM) for 1 h and then stimulated with IL-1β (1 ng/mL) for the indicated times. The same experiments were also performed including specific inhibitors of JNK (10 µM SP600125), p38 (10 µM SB202190), MEK1/2 (10 µM U0126) and NF-κB (5 µM Bay 11-7082). Cells in the negative control were treated with DMSO at the same concentrations as those present in quercetin or inhibitors. The levels of IL-6, IL-8, soluble ICAM-1 (sICAM-1) and MCP-1 were measured in samples of media using ELISA kits (R&D Systems, Minneapolis, MN, USA). The optical density of samples was measured spectrophotometrically with a microplate reader (Multiskan FC, Thermo) at 450 nm. All ELISAs were performed according to the manufacturers’ instructions [98].

### 4.5. Preparation of Cell Extracts and Western Blot Analysis

First, ARPE-19 cells were incubated with or without IL-1β at various concentrations (0.1, 1 or 2 ng/mL) for the indicated times. Second, cells were pretreated with quercetin (2.5 µM–20 µM) or inhibitors (10 µM U0126, 10 µM SP600125, 10 µM SB202190 or 5 µM Bay 11-7082) for 1 h before stimulation with 1 ng/mL IL-1β for either 1 h to measure the phosphorylation of protein or for 24 h to evaluate the ICAM-1 protein level. The concentrations of the choice of inhibitors had no toxic effects or changes in the cell viability on ARPE-19 cells, as tested for an LDH release test or a MTT assay (data not shown). The negative control was prepared as described in the previous section.

Proteins were extracted from ARPE-19 cells after being washed rapidly with ice-cold phosphate-buffered saline (PBS) and subsequently added to lysis buffer (25 mM NaCl (pH 7.4), 25 mM NaF, 25 mM Tris-HCl, 1 mM sodium vanadate, 25 mM sodium pyrophosphate, 2.5 mM ethylenediaminetetraacetic acid (EDTA), 2.5 mM ethylene glycol-bis(β-aminoethyl ether)-N,N,N’,N’-tetraacetic acid (EGTA), 0.05% (*v*/*v*) Triton X-100, 0.5% (*w*/*v*) sodium dodecyl sulfate (SDS), 0.5% (*w/v*) deoxycholate, 5 µg/mL aprotinin, 0.5% (*w*/*v*) NP-40, 5 µg/mL leupeptin and 1 mM phenylmethylsulfonyl fluoride (PMSF)). The resulting lysates were then centrifuged for 10 min at 15,000 rpm and 4 °C. A Pierce bicinchoninic acid (BCA) protein assay kit (Thermo Fisher Scientific, Rockford, IL, USA) was utilized to evaluate the protein concentration.

The same amount of protein (30 µg) from each sample was denatured, separated on a 10% gel for SDS polyacrylamide gel electrophoresis and transferred onto Immobilon-P transfer membranes (Millipore, Billerica, MA, USA), which were then blocked with the blocking buffer (Visual Protein, Taipei, Taiwan) for 60 min and incubated with a 1:1000 dilution of primary antibodies (anti-phospho-c-Jun, anti-phospho-ATF-2, anti-phospho-CREB, anti-phospho-ERK1/2, anti-phospho-p38, anti-phospho-JNK1/2, anti-phospho-IKKα/β, anti-phospho-NF-κB p65, anti-ICAM-1 and anti-GAPDH) at 4 °C overnight. Next, these membranes were washed with Tween-Tris-buffered saline (TTBS; 150 mM NaCl, 50 mM Tris-HCl, 0.05% (*v/v*) Tween 20, pH 7.4) and then incubated with anti-mouse or anti-rabbit horseradish peroxidase-conjugated secondary antibodies at a dilution of 1:10,000 for 1 h at room temperature. Finally, these membranes were washed four times for 15 min each with TTBS and incubated with enhanced chemiluminescence reagents to detect and quantify the specific protein using a ChemiDoc XRS+ system (Bio-Rad Laboratories, Inc., Hercules, CA, USA).

### 4.6. Total RNA Extraction and RT-qPCR

ARPE-19 cells were pretreated with or without 20 µM quercetin or inhibitors (5 µM Bay 11-7082, 10 µM SB202190, 10 µM U0126 or 10 µM SP600125) for 1 h before being stimulated with 1 ng/mL IL-1β for 4 h. The total RNA of ARPE-19 cells was extracted using TRIzol reagent (Sigma-Aldrich) as per the manufacturer’s protocol. The RNA concentration was then measured with a microspectrophotometer (Nano-100; Allsheng Instruments, Hangzhou City, Zhejiang, China). The 260/280 ratios of all samples are between 1.8–2. An iScript cDNA Synthesis Kit (Bio-Rad) was used to reverse transcribe total RNA into cDNA. Gene expression was quantified using an iQ™ SYBR Green Supermix kit (Bio-Rad Laboratories, Hercules, CA, USA) and a CFX connect Real-Time PCR Detection System (Bio-Rad).

A melting curve analysis was performed to verify the accuracy of the amplicon after the amplification program. The relative gene expression was estimated using the ΔΔCt method: β-actin expression served as an internal control and the ratio of the number of copies of the target gene mRNA to the number of copies of β-actin was calculated. All data are expressed as the fold-change relative to the mRNA level in the control cells. Each sample was run in triplicate. Primer Express software (PrimerQuest Tool, IDT, Inc., Coralville, IA, USA) was used to design the primers for qPCR to span exon–exon boundaries. The primers used for the target genes are listed in Table 1.

### 4.7. Immunofluorescence Staining

ARPE-19 cells were seeded into six-well culture plates with coverslips until they were 50–60% confluent and then pretreated with or without 1 ng/mL IL-1β for the indicated times (0, 5, 10, 15, and 30 min). Quercetin (20 µM) or an NF-κB inhibitor (5 µM Bay 11-7082) were added for 1 h before application of 1 ng/mL IL-1β for 10 min. Next, cells were fixed with 4% (*w/v*) paraformaldehyde for 15 min, permeabilized with 0.3% Triton X-100 for 1 min, blocked with PBS containing 5% (*w/v*) bovine serum albumin for 15 min and stained with an anti-NFκB p65 antibody overnight at 4 °C. The next day, the coverslips were treated with secondary antibody for 1 h at room temperature and mounted with aqueous mounting medium containing 4′,6-diamidino-2-phenylindole (Vector Laboratories, Burlingame, CA, USA). Cells were washed 2–3 times with PBS between each of the above experimental steps. The images were examined using a fluorescence microscope (Leica Microsystems, Wetzlar, Germany).

### 4.8. Monocyte Adhesion Assay

Before being stimulated with 1 ng/mL IL-1β for 24 h, ARPE-19 cells were pretreated with or without quercetin (10 or 20 µM) or inhibitors (5 µM Bay 11-7082, 10 µM SB202190, 10 µM SP600125 or 10 µM U0126) for 1 h. THP-1 cells were labeled with 5 µM calcein AM (a fluorescent dye) at 37 °C for 30 min in RPMI-1640 medium in the dark and then washed by centrifugation. Next, the labeled THP-1 cells (5 × 10^5^ cells/mL) were cocultured with ARPE-19 cells in plates for 1 h and washed gently three times with PBS to remove nonadherent THP-1 cells. Finally, the numbers of fluorescently labeled adherent THP-1 cells in five random fields were counted under a fluorescence microscope (Leica Microsystems).

### 4.9. Statistical Analysis

The intensity of the bands on the Western blotting and the numbers of fluorescently labeled adherent THP-1 cells in the monocyte adhesion assay were quantified using Image Lab software (Bio-Rad) and Image J software (W. Rasband, NIH, USA), respectively. All quantitative data are presented as the mean ± SD of at least three independent experiments. One-way analysis of variance followed by Tukey’s post hoc test using GraphPad Prism version 7 (GraphPad Software Inc., San Diego, CA, USA) was performed to identify the differences among multiple groups. The results were considered significant if *p* < 0.05.

## 5. Conclusions

**Figure 10:**
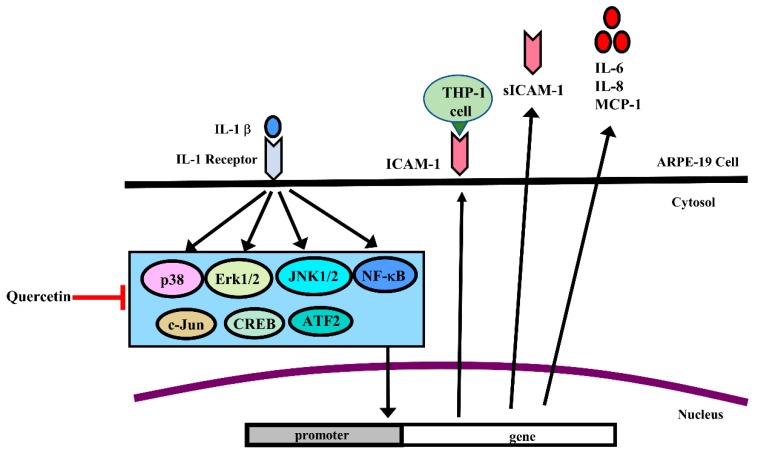
Schematic diagram of the signaling pathways involved in attenuation of IL-1β-induced inflammation by quercetin via downregulation of ICAM-1, sICAM-1, IL-6, IL-8 and MCP-1 expression in ARPE-19 cells. Quercetin attenuated ICAM-1, sICAM-1, IL-6, IL-8 and MCP-1 expression via the MAPK or NF-κB pathways in IL-1β-stimulated ARPE-19 cells.

The results of this study clearly demonstrated that the proinflammatory cytokine IL-1β significantly increased the protein and gene expression of ICAM-1, sICAM-1, IL-6, IL-8 and MCP-1 in ARPE-19 cells. We also provided evidence for the first time that quercetin markedly decreased the protein and gene expression of these cytokines and chemokines in IL-1β-stimulated ARPE-19 cells. Quercetin also inhibited signaling pathways associated with the inflammatory process, including phosphorylation of MAPKs, NF-κB p65, IKKα/β, c-Jun, CREB and ATF2, and blocked the translocation of NF-κB p65 into the nucleus (Figure 10). In conclusion, quercetin has the potential to ameliorate inflammatory responses in RPE cells and may serve as a therapeutic intervention for retinal inflammatory diseases such as AMD.

## Figures and Tables

**Table 1:** Primers used in RT-qPCR analyses of mRNA expression.

Gene	Primers	(5′-3′ Sequence)	GenBank Accession Number	Product Size (bp)
IL-6	Forward Reverse	TCGGTCCAGTTGCCTTCTC GAGGTGAGTGGCTGTCTGT	NM_000600	121
IL-8	Forward Reverse	GCAGAGGGTTGTGGAGAAGT TGGCATCTTCACTGATTCTTGG	NM_000584	90
MCP-1	Forward Reverse	GAATCACCAGCAGCAAGTGT GAGTGTTCAAGTCTTCGGAGTT	NM_002982	149
ICAM-1	Forward Reverse	ACCATCTACAGCTTTCCGGC CTGAGACCTCTGGCTTCGTC	NM_000201.2	55

## Abbreviations

AMD	age-related macular degeneration
ATF	activating transcription factor
ARPE-19 cells	human retinal pigment epithelial cells
CREB	cAMP response element-binding protein
DMSO	dimethyl sulfoxide
EDTA	ethylenediaminetetraacetic acid
ELISA	enzyme-linked immunosorbent assay
ERK	extracellular signal-regulated kinase
FBS	fetal bovine serum
GAPDH	glyceraldehyde 3-phosphate dehydrogenase
IκB	inhibitor of NF-κB
ICAM-1	intercellular adhesion molecule-1
IKK	inhibitor of nuclear factor κ-B kinase
IL-1β	interleukin-1β
IL-6	interleukin-6
IL-8	interleukin-8
JNK	c-Jun N-terminal kinase
MAPK	mitogen-activated protein kinase
MCP-1	monocyte chemoattractant protein-1
MMP	matrix metalloproteinase
MTT	3-(4,5-dimethylthiazol-2-yl)-2,5-diphenyltetrazolium bromide
NF-κB	nuclear factor κB
PBS	phosphate-buffered saline
SDS	sodium dodecyl sulfate
TNF-α	tumor necrosis factor-α
